# High Stubble Height Enhances Ratoon Rice Yield by Optimizing Light–Temperature Resource Utilization and Photothermal Quotient

**DOI:** 10.3390/plants14142222

**Published:** 2025-07-18

**Authors:** Yin Zhang, Tian Sheng, Liyan Shang, Beiyou Zhang, Long Jin, Fangfang Hou, Matthew Tom Harrison, Liying Huang, Zhaoqiang Jin, Xiaohai Tian, Ke Liu, Shijie Shi, Yunbo Zhang, Dayong Li

**Affiliations:** 1 Hubei Key Laboratory of Waterlogging Disaster and Agricultural Use of Wetland, College of Agriculture, Yangtze University, Jingzhou 434025, China; 2023710812@yangtzeu.edu.cn (Y.Z.); atianer2558@163.com (T.S.); lyshang9989@163.com (L.S.); 2023720891@yangtzeu.edu.cn (B.Z.); 2023710828@yangtzeu.edu.cn (L.J.); 2023720902@yangtzeu.edu.cn (F.H.); lyhuang8901@126.com (L.H.); jzq@yangtzeu.edu.cn (Z.J.); xiaohait@sina.com (X.T.); ke.liu@utas.edu.au (K.L.); shi.shijie@yangtzeu.edu.cn (S.S.); 2 Tasmanian Institute of Agriculture, University of Tasmania, Burnie 7320, Australia; matthew.harrison@utas.edu.au; 3 Jingzhou Agricultural Technology Promotion Center, Jingzhou 434020, China

**Keywords:** ratoon rice, stubble height, grain yield, photothermal quotient, light and temperature resources

## Abstract

Ratoon rice is a sustainable planting model, and its yield is closely linked to the light and temperature use efficiency. The photothermal quotient (PQ), a key parameter for evaluating the light and temperature use efficiency, significantly influences ratoon rice yield. However, research on how different stubble heights affect PQ and the utilization efficiency of light and temperature resources remains limited. Here, we conducted a two-year field experiment to investigate the radiation use efficiency (RUE), effective accumulated temperature use efficiency (TUE), PQ, interception percentage (IP), intercepted photosynthetically active radiation (IPAR), and total dry weight (TDW) of six ratoon rice varieties under two stubble height treatments (HS: high stubble, LS: low stubble) during the ratoon season. This study aimed to analyze how different stubble heights impact ratoon rice yield by evaluating light and temperature resource utilization efficiency and investigates the relationship between PQ and ratoon rice yield. The results showed that the HS treatment significantly increased ratoon season yield compared to LS treatment, with average yield increases of 21.2% and 28.1% in 2022 and 2023, respectively. This yield enhancement was attributed to improved TDW under HS treatment, driven by increased IP, IPAR, RUE, and TUE. Notably, PQ was significantly lower under HS than under LS treatment. This reduction was primarily attributed to the decreased duration available for light and heat accumulation, consequently lowering PQ. Correlation analysis revealed a significant positive association between main season yield and PQ, while ratoon season yield exhibited a negative correlation with PQ. In conclusion, the HS treatment increased IP and IPAR, enhanced TUE and RUE, and reduced PQ, collectively contributing to higher ratoon season yields. Importantly, our findings indicate that PQ can more effectively predict yield changes in the ratoon season under HS treatment, providing a theoretical basis for optimizing light and temperature resource utilization in ratoon rice.

## 1. Introduction

Rice (*Oryza sativa* L.) is a major cereal crop, and serves as a staple food for more than half of the world population [1]. China’s rice cultivation systems include single-cropping rice, double-cropping rice, and ratoon rice. Ratoon rice refers to a planting pattern in which, after the main season rice is harvested, the dormant buds on the rice stubble germinate into ratoon panicles under suitable cultivation conditions [2]. Ratoon rice is mainly cultivated in tropical and subtropical countries such as China, India, and countries in Southeast Asia. In China, the planting area of ratoon rice has reached 1.2 million hectares, and ratoon rice is mainly planted in southern provinces such as Hunan, Fujian, and Hubei [3]. In China, the average yields of the main season and the ratoon season for ratoon rice stand at approximately 9–10 t ha^−1^ and 5–6 t ha^−1^, respectively [4]. In Fujian province of China, the annual yield of ratoon rice can reach up to 15 t ha^−1^, and the highest yield of the ratoon season can reach 9.48 t ha^−1^ [5]. Given its low input cost, ratoon rice is capable of not only augmenting grain yield but also presenting excellent rice quality. As a result, the development of ratoon rice has been attracting growing attention [6,7].

However, several constrains have limited the further scaling up of mechanized ratoon rice. For example, the unfavorable growing environment, the lack of suitable varieties with high ratooning ability, the limited duration for rice growth, low use efficiency of light and temperature resources, and imperfect agricultural management practices are key factors responsible for the unstable ratoon season yield [8]. As for the agricultural management practices, the first and most important one is the unstable yield of the ratoon season due to rolling damage to stubbles during the mechanical harvesting of the main season [9]. Previous studies have shown that during the implementation of agricultural management practices, it was often necessary to consider the stubble height [10]. In general, the stubble height of the main season greatly influences ratoon season yield [11]. Previous studies have pointed out that in areas with limited light and temperature resources, a high stubble height (40–45 cm) can lead to the formation of more effective panicles due to the retention of more ratoon buds. Consequently, the yield of ratoon rice is effectively promoted to increase [12]. In regions with abundant light and temperature resources, a low stubble height (5–10 cm) is considered conducive to increasing the number of spikelets per panicle and the leaf area index, thereby increasing in the yield of ratoon rice is promoted [13]. Therefore, it is deemed of great significance to enhance the grain yield of ratoon rice by optimizing agricultural management practices. However, very few studies have explored the impact of stubble height on the yield of ratoon rice from the perspective of light and temperature utilization efficiency.

The selection of varieties serves as a crucial factor in influencing the grain yield of ratoon rice [11]. Various varieties accumulate discrepant amounts of light and temperature resources throughout their growth phases, thereby ultimately giving rise to disparities in grain yield. If the growth period of the main season rice is too long, it may lead to insufficient accumulated temperature in the ratoon season. On the contrary, if the growth period is too short, it may reduce the yield of the main season. Therefore, in the middle reaches of the Yangtze River region, the main season rice needs to be harvested before the mid-August to avoid the high temperature during the flowering period [3]. The ratoon season needs to have full heading before mid-September to avoid low temperature. The harvest period and growth period of the ratoon season depend on the temperatures in September and October. If the temperatures in these two months are high, the growth period will be prolonged, and the ratoon season can achieve high yields due to the accumulation of more heat [4]. In addition, for high yields, ratoon rice varieties with high yields in both the main season and the ratoon season should be selected for cultivation. Therefore, it is advisable to select high-yielding ratoon rice varieties with a moderate growth period for ratoon rice cultivation [14].

The efficient use of light and temperature resources is essential for crops to achieve high yields. Effective accumulated temperature (EAT, °C d) and solar radiation (SR, MJ m^−2^) significantly influence the growth duration and yield [15]. EAT represents the cumulative thermal energy required for a crop to complete a specific growth stage, calculated as the sum of daily temperatures above a defined biological threshold. SR, as the primary energy source for photosynthesis, drives the formation of crop yields. Radiation use efficiency (RUE, g MJ^−1^) is the ratio of total dry weight (TDW) to the intercepted photosynthetically active radiation (IPAR) in the growth stage of crop plants [16]. RUE is defined as the amount of biomass accumulated per unit solar radiation intercepted [17]. It is estimated that rice yield could be increased by approximately 20% if RUE is enhanced by 1% [18]. Grain yield depends on three main factors: light interception, RUE, and harvest index. Effective accumulated temperature use efficiency (TUE) is the ratio of effective accumulated temperature during the whole growth period to total accumulated temperature in the growth stage of crop plants, and there is a positive correlation between TUE and rice yield [19]. Nix first proposed the ratio of the daily solar radiation to effective accumulated temperature referred to as photothermal quotient (PQ, MJ m^−2^ d^−1^ °C^−1^) [20]. Generally, higher PQ values prolong the duration of photosynthetic activity in rice and increase the interception of photosynthetically active radiation, promoting spikelet formation, resulting in higher yields [21]. The PQ can be used to predict the impact of meteorological factors on the yield potential of crops [22]. Some studies have found that there is a linear relationship between the PQ from 20 days before the flowering of wheat to 10 days after the flowering and the kernels per spike. This indicates that the PQ can predict the yield of wheat quite well [23]. However, the research on PQ in rice is relatively scarce. Chakraborty et al., (2018) indicated that aboveground biomass and panicle weight showed a linear function of the PQ in rice [24]. In addition, previous studies have shown that PQ at the panicle initiation stage significantly and positively affected the grain yield and it can predict rice yield through temperature and solar radiation [21,25]. However, less research has been performed on stubble height on PQ and yield in ratoon rice systems. Therefore, we further explored the relationship between PQ and rice yield based on previous studies.

Previous studies mainly focused on investigating the ratooning ability to clarify the impact of stubble height on the yield of ratoon rice. However, studies on how the stubble height affects the yield of ratoon rice from the perspective of light and temperature resource utilization are relatively scarce. Consequently, the specific objectives of this study were to (1) evaluate the effect of the different stubble heights on ratoon rice yield from the perspective of light and temperature resource utilization; (2) investigate the relationship between PQ and ratoon rice yield. This study aims to provide a reference for ratoon rice production.

## 2. Results

### 2.1. Grain Yield

**Figure 1:**
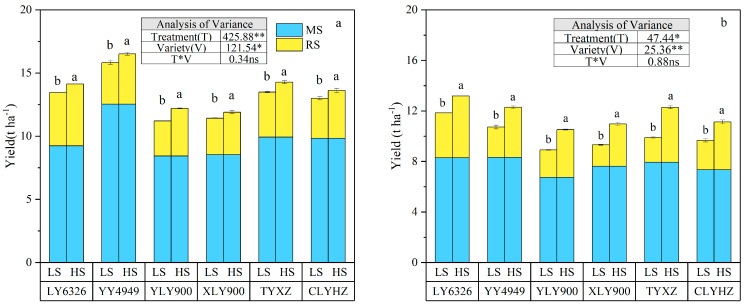
The annual yields of different varieties under different stubble treatments in 2022 (**a**) and 2023 (**b**). Within each column, different letters indicate significant differences in the yields of the ratoon season among different varieties according to LSD (0.05). Ns, non-significant; ** significant at *p* < 0.01; * significant at *p* < 0.05. MS, main season; RS, ratoon season; LS, low stubble; HS, high stubble.

All main season yields were higher than ratoon season yields in 2022 and 2023 (Figure 1). The annual yield was significantly affected by stubble treatment and variety (*p* < 0.05). Compared to the low stubble (LS) treatment, the annual yield of six ratoon rice varieties was significantly increased under the high stubble (HS) treatment, (*p* < 0.05). In 2022, YY4949 has the highest annual yield of 16.5 t ha^−1^. In terms of the main season yield, in both 2022 and 2023, YY4949 had the highest yield of 12.5 t ha^−1^ and 8.3 t ha^−1^, respectively. For the ratoon season, the HS treatment significantly increased ratoon season yield (*p* < 0.05). Specifically, compared to the LS treatment, the average yields in the ratoon season were increased by 21.3% and 40.2% under the HS treatment in 2022 and 2023, respectively.

### 2.2. EAT, SR, and PQ of Different Ratoon Rice Varieties

**Figure 2:**
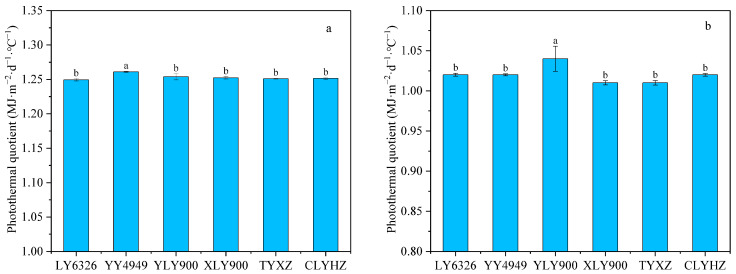
The photothermal quotient of the main season for different varieties in 2022 (**a**) and 2023 (**b**). Within each column, different letters indicate significant differences according to LSD (0.05).

**Figure 3:**
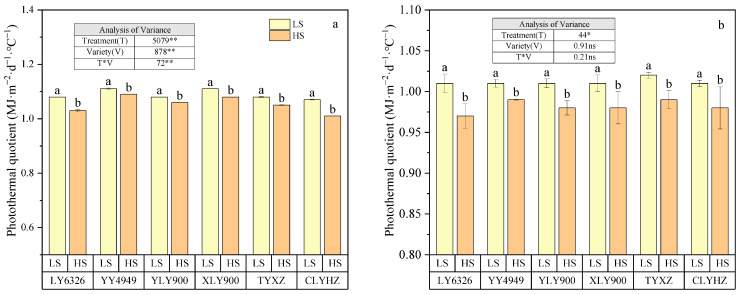
The photothermal quotient of the ratoon season for different varieties under different stubble treatments in 2022 (**a**) and 2023 (**b**). Within each column, different letters indicate significant differences according to LSD (0.05). Ns, non-significant; ** significant at *p* < 0.01; * significant at *p* < 0.05. LS, low stubble; HS, high stubble.

In 2022 and 2023, the EAT and SR in the ratoon season was significantly higher under the LS treatment than those under the HS treatment (Table 1, *p* < 0.05). This indicates that the growth period of rice was shortened under the HS treatment, leading to reduced accumulation of SR and EAT, which went against the common sense that the high SR and EAT were beneficial to the grain filling and yield formation. The reason for this result was that a high SR and EAT tended to be accompanied by a higher temperature, and the latter have negative influences on grain yield. In 2022 and 2023, the PQ of the main season was 17.1% and 1.9% higher than the ratoon season, respectively. PQ of YY4949 in the main season was significantly higher than other varieties, reaching 1.26 MJ m^−2^ d^−1^ °C^−1^ in 2022 (Figure 2, *p* < 0.05). The stubble treatment significantly affected the PQ of the ratoon season (Figure 3, *p* < 0.05). Specifically, the HS treatment reduced the PQ. Compared to the LS treatment, the PQ of the ratoon season was significantly reduced by 2.9% and 2.1%, under the HS treatment in 2022 and 2023, respectively.

### 2.3. TUE, TDW, IP, IPAR, and RUE of Different Ratoon Rice Varieties

The treatment and variety, as well as their interactions, had an extremely significant effect on TDW in the ratoon season and the annual period (Table 2 and Table 3, *p* < 0.01). In the ratoon season, the TDW was significantly increased by 29.5% and 48.1%, under the HS treatment in 2022 and 2023, respectively. Similarly, the interception percentage (IP) and IPAR were significantly increased by 34.6% and 28.7%, under the HS treatment in 2023, respectively (*p* < 0.05). The interaction between variety and stubble treatment had a significant effect on the IP in the ratoon season in 2023 (*p* < 0.05).

In 2022 and 2023, the TUE and RUE in both the ratoon season and the annual period were significantly affected by the variety and treatment (*p* < 0.05). Meanwhile, in both the ratoon season and the annual period, the TUE was significantly affected by the interaction between variety and stubble treatment, but the RUE was not significantly affected (*p* < 0.05). In the ratoon season, both the TUE and RUE were significantly enhanced by 3.3% and 17.3%, under the HS treatment in 2023, respectively (*p* < 0.05).

**Figure 4:**
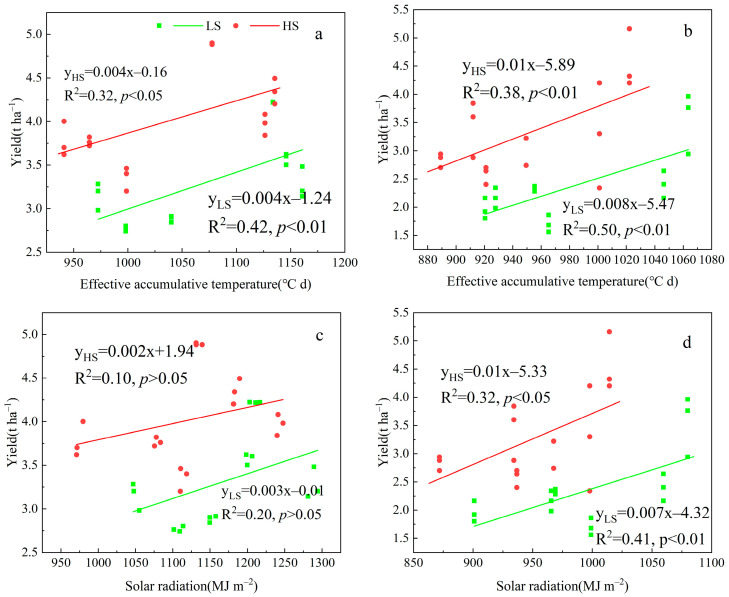
Correlation analysis of the yield in the ratoon season with effective accumulated temperature and solar radiation under different stubble treatments in 2022 ((**a**,**c**), respectively) and 2023 ((**b**,**d**), respectively). LS, low stubble; HS, high stubble. The green solid line represents the low stubble treatment, and the red solid line represents the high stubble treatment.

Since stubble treatment is carried out after the main season of rice, it primarily affects the yield, light, and temperature resources of the ratoon season, with no impact on those of the main season. Therefore, in Figure 4, we highlight the correlation between ratoon season yield of different rice varieties with effective accumulated temperature and solar radiation. The yield in the ratoon season showed a significant positive correlation with the EAT and SR (Figure 4, *p* < 0.05). Under the LS treatment, the correlation between the yield in the ratoon season and the EAT and SR was stronger. Specifically, regarding EAT, the correlation coefficients were 0.64 and 0.71 in 2022 and 2023 under the LS treatment, respectively. Moreover, regarding SR, the correlation coefficients were 0.44 and 0.64 in 2022 and 2023 under the low stubble treatment, respectively.

**Figure 5:**
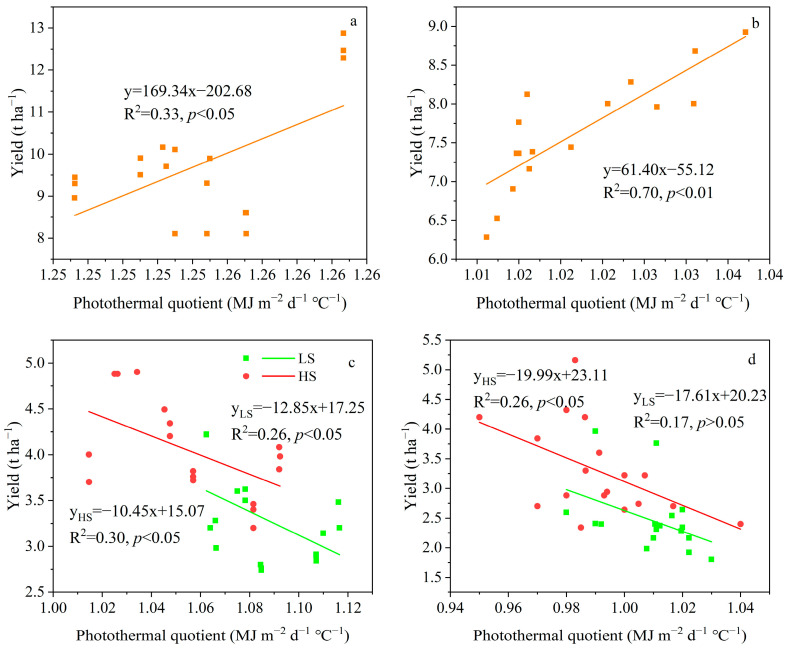
Correlation analysis of the yield of the main season with the photothermal quotient in 2022 (**a**) and 2023 (**b**). Correlation analysis of the yield of the ratoon season of different varieties with the photothermal quotient under different stubble treatments in 2022 (**c**) and 2023 (**d**). LS, low stubble; HS, high stubble. The green solid line represents the low stubble treatment, and the red solid line represents the high stubble treatment.

There was a significant positive correlation between the yield of the main season of different ratoon rice varieties and the PQ (*p* < 0.05). The correlation coefficients in 2022 (Figure 5a) and 2023 (Figure 5b) were 0.58 and 0.84, respectively. A negative correlation between the yield of the ratoon season and the PQ was shown. Specifically, in 2022 (Figure 5c) and 2023 (Figure 5d), under the HS treatment, the correlation coefficients were −0.55 and −0.51, respectively. The above results indicate that increasing the PQ is conducive to increasing the yield of the main season, while the converse is observed under stubble treatment. This indicates that PQ demonstrates a more robust predictive capacity for fluctuations in regeneration season yields.

**Figure 6:**
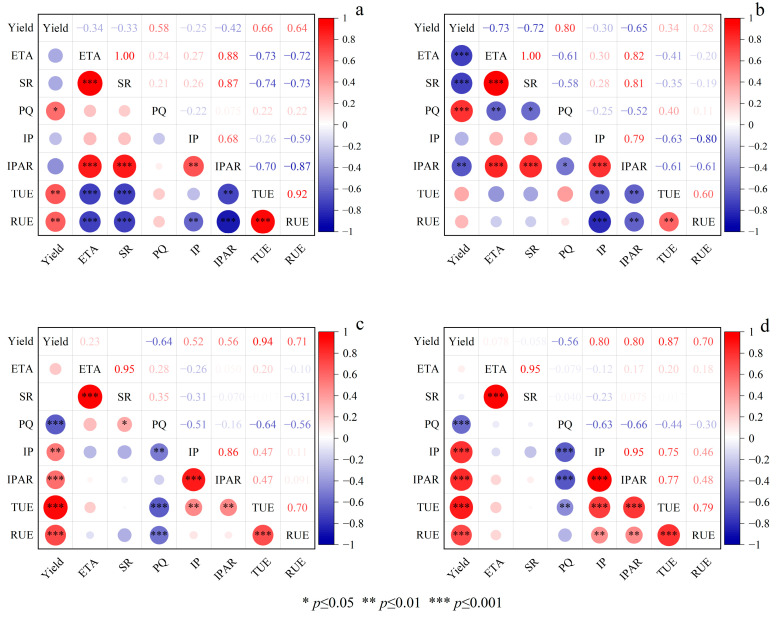
Correlation analysis of the yield of the main season with light and temperature resources in 2022 (**a**) and 2023 (**b**). Correlation analysis of the yield in the ratoon season under different stubble treatments with temperature and light resources in 2022 (**c**) and 2023 (**d**). EAT, effective accumulated temperature; SR, solar radiation; PQ, photothermal quotient; IP, interception percentage; IPAR, intercepted radiation; RUE, radiation use efficiency; TUE, effective accumulated temperature use efficiency.

Correlation analysis of the yield of the main season with light and temperature resources in 2022 and 2023. In 2022 (Figure 6a), an extremely significant positive correlation between the yield of the main season and the TUE, RUE, and PQ was shown, with correlation coefficients of 0.66, 0.64, and 0.68, respectively (*p* < 0.01). In 2023 (Figure 6b), an extremely significant positive correlation between the yield of the main season and the PQ was shown, with a correlation coefficient of 0.80 (*p* < 0.001).

Correlation analysis of the yield in the ratoon season under different stubble treatments with light and temperature resources. In 2022 (Figure 6c) and 2023 (Figure 6d), it was shown that there was an extremely significant positive correlation between the yield in the ratoon season and the TUE (r = 0.94), RUE (r = 0.71), and IPAR (r = 0.56). In 2023 (Figure 6d), it was shown that there was an extremely significant positive correlation between the yield in the ratoon season and the TUE (r = 0.87), RUE (r = 0.7), and IPAR (0.8) (*p* < 0.001). An extremely significant negative correlation was shown between the yield in the ratoon season and the PQ (*p* < 0.001), with correlation coefficients of −0.64 and −0.56 in 2022 and 2023, respectively.

## 3. Discussion

Stubble height is a crucial agronomic practice that greatly determines the grain yield of ratoon rice and different stubble management practices have varying impacts on ratoon rice yield [26]. Different scholars hold different views on the appropriate stubble height for ratoon rice. Petroudi et al., (2011) concluded that the yield in the ratoon season can be increased by low stubble height [27]. This is consistent with previous research findings [28,29]. For varieties with a strong ability to regenerate from the basal nodes of the stubble, it is recommended to harvest the main crop at a low cutting height. In contrast, for varieties that mainly regenerate from panicles in the upper nodes, increasing the stubble height significantly improves the grain yield of the ratoon season [3]. Low stubble height prolonged the growth duration, slowed down the rate of leaf senescence, decreased the panicle number, and increased the spikelets panicle^−1^, total spikelets, and leaf area index [13]. Harrell et al. (2009) indicated that when the initial stubble height is reduced from 40 to 20 cm, the growth of the ratoon season is altered by shifting the panicle point of origin during the early growth period and delaying maturity, and the yield advantage was associated with the increased weight of the basal panicles when the main season was harvested at 20 cm [30]. In contrast, Li et al., (2021) reported that the yield of the ratoon season with a high stubble height (40 cm) was 9.9% higher than that of the medium stubble height (20 cm) and 16.3% higher than that of the low stubble height (15 cm) [31]. Our results revealed that the HS treatment (40 cm) significantly increased the yield of the ratoon season by 21.2% to 40.2%, compared with the LS treatment (25 cm), which is consistent with the results of Torres’ research [32]. In this study, the increase in yield resulting from high stubble is attributed to increased IP, IPAR, RUE, and TUE (Table 2 and Table 3). This increase aligns with findings by Nakano et al., (2021), where high stubble also led to enhanced IP [12]. In addition, some scholars have pointed out that the stubble height for the ratoon season should depend on the local climatic conditions. In regions with abundant light and temperature resources, a low stubble height can be chosen to fully use these resources. In regions with insufficient light and temperature resources, scholars recommend a stubble height of around 40 to 45 cm to ensure safe panicle emergence, grain filling, and enhance the regeneration rate [29,33]. Research on the utilization efficiency of light and temperature resources under stubble height in ratoon rice is relatively scarce. Therefore, further research will be conducted to investigate the impact of stubble height on the TUE, RUE, and related parameters of ratoon rice in the future.

Another important factor affecting the yield of ratoon rice is the choice of rice variety. Different rice varieties vary in use efficiency of light and temperature resources, which lead to differences in yield potential. Our study indicated that the annual yield of YY4949 is the highest, reaching 16.5 kg·ha^−1^ under the high stubble in 2022, which was attributed to RUE and IPAR (Table 2), which was consistent with the study of Huang et al. (2019) on the reasons for the high yield of YY4949 [34]. The annual yield of LY6326 is the highest, reaching 12.8 kg·ha^−1^ under the high stubble in 2023, which was attributed to IP, RUE, and TDW (Table 3), which was consistent with the study of Qi et al. (2024) on the reasons for the high yield of LY6326 [35]. This suggests that the yield of the ratoon season is unstable and susceptible to environmental varieties and management factors [4]. YY4949 and LY6326 are a hybrid variety. Previous studies found the hybrids usually have higher yield potential of ratoon rice than inbred varieties [36]. More importantly, two varieties including YY4949 and LY6326 were identified to have higher grain yield than most of other varieties in both the main season and ratoon season (Figure 1). These results suggested that YY4949 and LY6326 could be the suitable varieties for achieving high grain yield in ratoon rice in central China. This suggests that the yield of the ratoon season is unstable and susceptible to environmental varieties and management factors. Therefore, future research on ratoon rice should focus on breeding rice varieties with high light utilization efficiency.

The photothermal quotient (PQ) integrates the comprehensive impacts of solar radiation and temperature on the growth and development of crops and it has been widely applied to predict crop yields [37]. Previous studies have shown that crop yield is positively correlated with the PQ [38]. Porker et al. (2025) found that during the reproductive growth stage of barley, the correlation between yield and the PQ is stronger [39]. Previous studies have shown that PQ at panicle initiation stage significantly and positively affected the grain yield [25]. We investigated the relationship between PQ and the yield of ratoon rice. This study reveals that main season yield is significantly positively correlated with PQ, whereas ratoon season yield shows a negative correlation with PQ. This result is attributed to the accumulation of both EAT and SR, leading to an increase in PQ in the main season. Meanwhile, our results showed that PQ was positively correlated with TUE and RUE, with yield formation driven by TUE and RUE in the main season (Figure 6a,b). In ratoon season, the intensity of the SR has weakened, and the accumulation rate of SR was lower than that of EA, which resulted in a decrease in PQ. Our results showed that PQ was negatively correlated with IP, IPAR, TUE, and RUE, with yield formation driven by IP, IPAR, TUE, and RUE (Figure 6c,d). In this study, the PQ is reduced under the HS treatment, compared with the LS treatment. The reason for this result is that the growth period was shortened under the HS treatment, reducing EAT and SR by 41.4 °C d and 82.7 MJ m^−2^ in 2022, respectively (Table 1), lowering PQ. However, HS retains more functional leaves, increasing IP, IPAR, RUE, and TUE by 34.6%, 4.6%, 17.4%, and 3.3% in 2023, respectively (Table 3). In short, shorter growth periods under HS reduce PQ, but enhanced IP, IPAR, RUE, and TUE dominate yield formation. Correlation analysis indicates that the linear fit between ratoon season yield and PQ is stronger under the HS treatment compared to the LS treatment, with correlation coefficients of −0.55 and −0.51 in 2022 and 2023, respectively. Therefore, it indicates that the PQ can effectively predict the changes in the yield of the ratoon season under the HS treatment. To sum up, the PQ is positively correlated with the yield of the main season and negatively correlated with the yield of the ratoon season. HS treatment reduces the PQ and increases the yield. At present, the PQ is widely used to predict the potential yield changes of wheat, but there are few studies in ratoon rice. Facing the changeable climatic conditions, future research needs to combine field experiments and model simulations to further explore the relationship between the yield of ratoon rice and the PQ. This will be beneficial for predicting the yield changes of ratoon rice through temperature and light resources.

Temperature and solar radiation are critical limiting factors influencing rice growth, development, and yield formation. Effective accumulated temperature (EAT) represents the cumulative thermal energy required for a crop to complete a specific growth stage or its entire life cycle. Previous studies have shown that the grain yield was determined by the EAT before the heading stage [40]. A sufficient solar radiation supply is the basis for grain-filling after heading, which is significantly and positively correlated with rice yield [16]. Radiation use efficiency (RUE) is one of the three important resource use efficiencies in crops and is an essential indicator for measuring crop yield. Some studies indicate that biomass production can be increased by increasing RUE [34]. Thus, it is crucial to improve the use efficiency of light and temperature resources for rice by optimizing agronomic measures to increase rice yield [41]. However, currently, there are few reports on the research of the use characteristics of light and temperature resources by ratoon rice under stubble treatments. Therefore, this study explored the effects of two stubble treatments on the EAT and the SR of different rice varieties. The study found that, compared with the HS treatment, the LS treatment significantly increased the EAT and the SR of ratoon rice (Table 1). This might be related to the extension of the growth period of the ratoon season under the LS treatment [10]. In actual production, optimizing cultivation management measures have been adopted to improve the RUE and TUE of rice, which is a way to achieve high yields [42]. Our results revealed that the TUE and RUE in the ratoon season was significantly increased under the HS treatment. It is shown by the correlation analysis that a significant positive correlation exists between the yield of the ratoon season and the IPAR, RUE, and RUE. Meanwhile, the IPAR, TUE, and RUE in the ratoon season are significantly improved under the HS treatment. The above results indicate that implementing the HS treatment is beneficial to increasing the yield of the ratoon season.

However, our study also has some limitations. The contributions of TUE, IPAR, and RUE to aboveground biomass accumulation and yield under different stubble heights treatments were only assessed in one ecological region, limited number of varieties, years, and absence of detailed physiological or biochemical measurements (carbohydrate remobilization). Therefore, further research is needed to confirm these contributions of ratoon rice across multiple years, and multiple ecological regions under different stubble heights treatments. In addition, targeted breeding for traits such as ratoon bud vigor, stubble node survival, or carbohydrate remobilization is recommended [43,44]. Moreover, we propose the development of integrated crop-climate models using PQ, IPAR, and RUE for yield forecasting under climate change [45].

## 4. Materials and Methods

### 4.1. Experimental Site and Test Material

**Figure 7:**
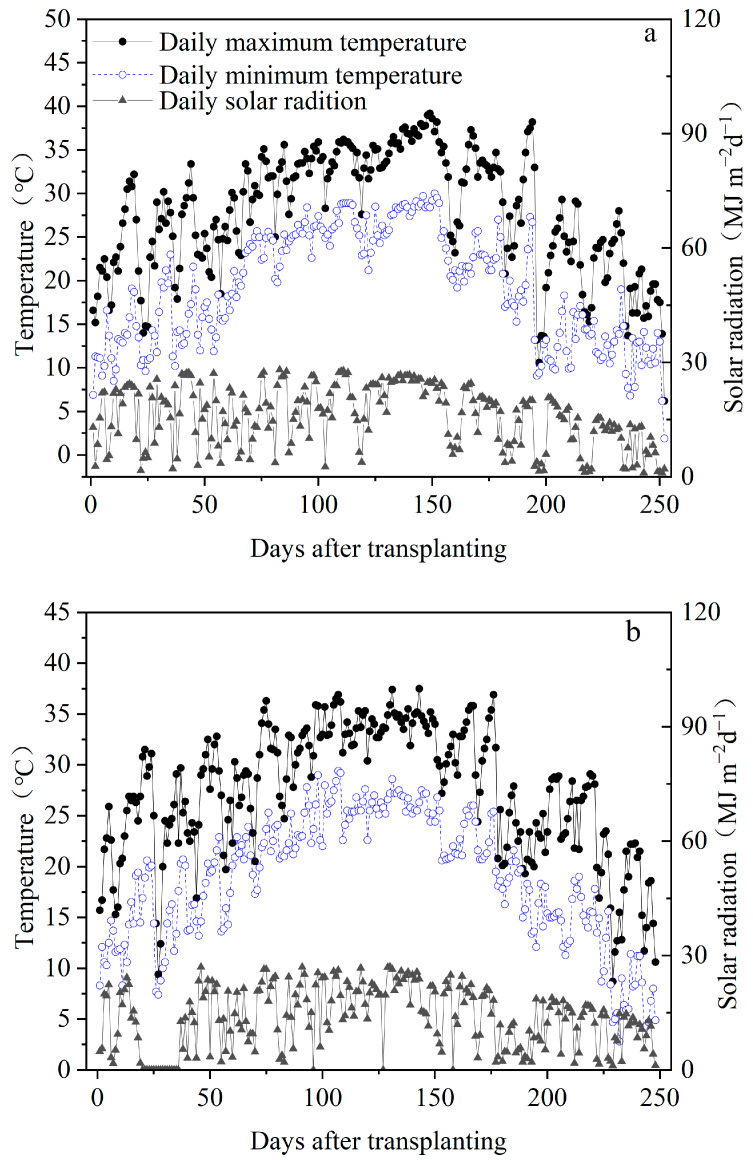
Daily maximum and minimum temperature and solar radiation during rice growing season from transplanting to maturity in 2022 (**a**) and 2023 (**b**), in Jingzhou, Hubei Province, China.

From 2022 to 2023, a two-year field study was conducted at Yangtze University’s experimental farm in Jingzhou, Hubei Province, China (112°31′ E, 30°21′ N). The daily maximum, minimum temperature, and solar radiation during the rice growing season were measured by meteorological stations near the experimental field. During the growth period of rice in 2022, the average daily maximum temperature, average daily minimum temperature, and average daily solar radiation were 29.2 °C, 20.5 °C, and 17.1 MJ m^−2^ d^−1^, respectively. During the growth period of rice in 2023, the average daily maximum temperature, average daily minimum temperature, and average daily solar radiation were 28.5 °C, 20.0 °C, and 14.0 MJ m^−2^ d^−1^, respectively (Figure 7). Before the experiment, soil samples from the top 20 cm were gathered to assess soil qualities. Soil samples were collected from the four corners and the center of each treatment plot. The yearly soil characteristic value was then calculated using the average of the four soil samples. The soil in this area is gray fluvo-aquic soil, with the pH of 6.7, 22.7 g kg^−1^ of organic matter, 1.9 g kg^−1^ of total nitrogen, 25.6 mg kg^−1^ of readily available phosphorus, and 126.9 mg kg^−1^ of available potassium.

Liangyou6326 (YL6326), Yongyou4949 (YY4949), Y-liangyou900 (YLY900), Xiangliangyou900 (XLY900), C-liangyouhuazhan (CLYHZ), and Taoyouxiangzhan (TYXZ) were used as test materials in the experiment. The growth periods of these six varieties are moderate, and they are widely planted in the southern regions of China. The specific information of the varieties is shown in Table 4.

### 4.2. Experimental Design and Crop Management

The experiment adopted a split-plot design, with the stubble height as the main plot and the variety as the sub-plot. The main plots consisted of two stubble treatments: the low stubble treatment (LS: 25 cm) and the high stubble treatment (HS: 40 cm). Pre-germinated seeds were planted at a rate of 25 g m^−2^ in a seedbed, and seedlings were transplanted to field plots at 32 days old, with hill spacings of 30 cm × 14 cm and two seedlings per hill. The management of crops adhered to accepted cultural practices. Transplanting dates were 28 April 2022 and 28 April 2023, respectively. The experiment was designed with three replicates per variety.

A total of 225 kg N ha^−1^ of N fertilizer as urea was applied at the basal (1 day before transplanting) to the main season, tillering (7 days after transplanting), and panicle initiation stage at a ratio of 3:3:4. Phosphorus (45 kg P ha^−1^ as calcium superphosphate) and potassium (100 kg K ha^−1^ as potassium chloride) were applied in the same amount in each plot. Phosphorus was applied as basal fertilizer and potassium was split equally between the basal and panicle initiation stages. Meanwhile, 75 kg N ha^−1^ and 45 kg K_2_O ha^−1^ were applied approximately 15 d after heading in the main season to improve the sprouting of regenerated buds. Second, 75 kg N ha^−1^ and 45 kg K_2_O ha^−1^ were applied and tiller-promoting fertilizers applied 2–5 d after harvesting the main season, significantly enhancing regeneration ability and ratoon season grain yield.

Immediately after the main season is harvested, water is put on to prevent the stumps from dying in the sun due to high temperatures. Pesticides were extensively used to control insects and prevent yield and biomass losses.

At maturity, the grain yield was determined from a 5 m^2^ sampling area in the center of each plot and adjusted to 14% moisture content in 2022 and 2023. An additional six hills plants from each plot were sampled to determine the total dry weight according to the method of Huang [34].

### 4.3. Measurement of EAT and TUE

Liu’s method [46] was employed to determine the effective accumulated temperature (EAT) and effective accumulated temperature use efficiency (TUE). Specifically, the EAT of rice was the sum of the temperatures ≥ 10 °C during the growing season.

The total accumulated temperature is the accumulation of the daily average temperature with the temperature being ≥10 °C during the growth period of rice. Thus, the TUE (%) = (effective accumulated temperature during the whole growth period/total accumulated temperature) × 100%.

### 4.4. Measurement of SR, and RUE

The SunScan Canopy Analysis System was used to measure canopy light interception between 11.00 and 13.00 h at MT, PI, HD, and MA (Delta-T Devices Ltd., Burwell, UK). Each plot’s canopy light intensity was measured by setting the light bar halfway between two rows and just over the water’s surface. Both three readings between rows and three readings within rows were taken. At the same time, the amount of incoming light was measured. Calculated as [100 (incoming light intensity-light intensity inside the canopy)/incoming light intensity], this is the intercepted percentage of incoming light intensity by the canopy. IPAR was estimated to account for 0.45% of the overall solar radiation [47].

The IPAR during each development stage was calculated using the average canopy light absorption and the total solar radiation absorbed throughout the stage [1/2 (beginning of the development stage canopy light interception and ending of the growth stage canopy light interception) cumulative radiation exposure during the growth phase]. The intercepted radiation from each growth phase was aggregated to determine the IPAR for the entire growing season. The RUE during the entire growth stage was calculated using the sum of the IPAR during each development stage. The RUE was calculated using the above-ground TDW to IPAR ratio for the entire growing season. A Vantage Pro2 weather station (Davis Instruments Corp., Hayward, CA, USA) was used to record the sun radiation and lowest and maximum temperatures every day.

### 4.5. Measurement of Photothermal Quotient

Fischer’s method [20] was employed to determine the photothermal quotient (PQ). PQ = DSR/(T − T_0_), where DSR is daily average solar radiation, T and T_0_ represent the daily average temperature and the base temperature, which is set at 10 °C for rice, respectively.

### 4.6. Data Analysis

Analysis of variance was performed with Statistic 9.0 (Analytical Software, Tallahas-see, FL, USA), and means were compared based on the least significant difference (LSD) test at the 0.05 probability level. All figures were constructed using Origin 2025.

## 5. Conclusions

In this experiment, we investigated the effects of two stubble heights on ratoon rice yield and light and temperature resource use efficiency. Our findings indicate that, compared with the LS treatment, the HS treatment significantly increased the yield of the ratoon season. This result can be attributed to the enhanced total dry weight brought about by increased IP, IPAR, RUE, and TUE. Additionally, the yield of the main season shows a significant positive correlation with the PQ. In contrast, the yield of the ratoon season exhibits a negative correlation with the PQ. Under the HS treatment, the PQ was reduced, leading to a significant increase in ratoon season yield. Importantly, our study indicated that changes in ratoon season yield under the HS treatment can be more effectively predicted by the PQ.

## Figures and Tables

**Table 1:** The effective accumulated temperature and solar radiation of different ratoon rice varieties in 2022 and 2023.

Year	Variety	Treatment	MS		RS		TS	
			EAT	SR	EAT	SR	EAT	SR
			(°C d)	(MJ m^−2^)	(°C d)	(MJ m^−2^)	(°C d)	(MJ m^−2^)
2022	LY6326	LS	1860.5	2320.5	1135.6	1229.2	2996.1	3549.7
		HS	1860.5	2320.5	1101.5	1130.4	2962.0	3451.0
	YY4949	LS	1829.4	2309.9	1262.2	1409.4	3091.6	3719.3
		HS	1829.4	2309.9	1214.3	1326.1	3043.7	3636.0
	YLY900	LS	1939.8	2438.5	1103.9	1197.5	3043.7	3636.0
		HS	1939.8	2438.5	1053.3	1113.3	2993.1	3551.8
	XLY900	LS	1985.6	2491.6	1095.0	1212.3	3080.6	3703.9
		HS	1985.6	2491.6	1058.1	1144.4	3043.7	3636.0
	ZYXZ	LS	2030.4	2543.1	1013.3	1092.6	3043.7	3635.7
		HS	2030.4	2543.1	962.7	1008.4	2993.1	3551.5
	CLYHZ	LS	2053.2	2568.7	956.3	1019.5	3009.5	3588.3
		HS	2053.2	2568.7	927.9	941.5	2981.1	3510.2
	Average	LS	1949.8	2445.4	1094.4 a	1193.4 a	3044.2 a	3638.8 a
		HS	1949.8	2445.4	1053.0 b	1110.7 b	3002.8 b	3556.1 b
2023	LY6326	LS	2099.5	2143.4	899.7	908.7	2999.2	3052.1
		HS	2099.5	2143.4	868.3	842.3	2967.8	2985.7
	YY4949	LS	1996.6	2039.8	1044.0	1055.0	3040.6	3094.8
		HS	1996.6	2039.8	1002.6	992.6	2999.2	3032.4
	YLY900	LS	2037.2	2089.0	1026.0	1033.9	3063.2	3122.9
		HS	2037.2	2089.0	980.8	961.2	3018.0	3050.2
	XLY900	LS	2138.1	2169.6	965.3	975.0	3103.4	3144.5
		HS	2138.1	2169.6	902.5	884.4	3040.6	3054.0
	ZYXZ	LS	2138.1	2169.6	946.7	965.6	3084.8	3135.2
		HS	2138.1	2169.6	930.8	921.5	3068.9	3091.1
	CLYHZ	LS	2176.3	2213.5	919.8	929.0	3096.1	3142.5
		HS	2176.3	2213.5	898.6	880.6	3074.9	3094.1
	Average	LS	2097.6	2137.5	966.9 a	977.9 a	3064.6 a	3115.4 a
		HS	2097.6	2137.5	930.6 b	913.8 b	3028.2 b	3051.2 b

MS, main season; RS, ratoon season; TS, the total of the two seasons; EAT, effective accumulated temperature (°C d); SR, solar radiation (MJ m^−2^). Within each column, means followed by the different letters are significantly different according to LSD (0.05).

**Table 2:** Effective accumulated temperature use efficiency, radiation use efficiency, and their related parameters for different ratoon rice varieties in 2022.

Variety	Treatment	TUE (%)		TDW (g m^−2^)			IP (%)		IPAR (MJ m^−2^)			RUE (g MJ^−1^)		
		MS	RS	MS	RS	TS	MS	RS	MS	RS	TS	MS	RS	TS
LY6326	LS	63.3	61.4 b	2211.7	936.0 b	3147.7 b	86.9	73.0 b	827.5	396.2 a	1223.7 a	2.6	2.4 b	2.6 b
	HS	63.3	62.2 a	2211.7	1158.6 a	3370.3 a	86.9	80.4 a	827.5	406.7 a	1234.2 a	2.6	2.9 a	2.7 a
YY4949	LS	64.7	55.7 b	2397.4	732.0 b	3129.4 b	86.3	60.2 b	837.1	343.6 b	1180.6 b	2.8	2.2 b	2.7 a
	HS	64.7	57.0 a	2397.4	997.5 a	3394.9 a	86.3	76.6 a	837.1	426.9 a	1264.0 a	2.8	2.6 a	2.7 a
YLY900	LS	65.0	55.0 b	2340.4	718.3 b	3058.7 b	90.0	74.9 a	925.8	372.5 a	1298.3 b	2.5	1.9 b	2.4 b
	HS	65.0	56.3 a	2340.4	865.8 a	3206.2 a	90.0	78.6 a	925.8	374.9 a	1300.8 a	2.5	2.3 a	2.5 a
XLY900	LS	63.0	56.7 b	2072.3	713.6 b	2785.9 b	89.2	63.8 b	909.5	326.1 b	1235.5 b	2.3	2.2 b	2.3 a
	HS	63.0	57.1 a	2072.3	954.1 a	3026.5 a	89.2	78.0 a	909.5	397.0 a	1306.5 a	2.3	2.4 a	2.3 a
TYXZ	LS	54.6	68.1 b	2251.9	889.4 b	3141.3 b	88.6	66.8 b	776.9	354.6 b	1131.5 b	2.9	2.5 b	2.8 b
	HS	54.6	71.7 a	2251.9	1245.6 a	3497.5 a	88.6	82.9 a	776.9	434.6 a	1211.5 a	2.9	2.9 a	2.9 a
CLYHZ	LS	60.0	61.3 b	2281.5	695.3 b	2976.8 b	95.1	63.8 b	919.3	300.1 b	1219.5 b	2.5	2.3 b	2.4 b
	HS	60.0	62.0 a	2281.5	843.6 a	3125.0 a	95.1	75.4 a	919.3	341.9 a	1261.3 a	2.5	2.5 a	2.5 a
Variety	LY6326	63.3 C	61.8 B	2211.7 D	1047 A	3259 AB	86.9 CD	76.7 A	827.5 B	401.4 A	1229.0 BC	2.6 B	2.6 A	2.7 B
	YY4949	64.7 B	56.4 D	2397.4 A	864.7 B	3262.1 AB	86.3 D	68.4 C	837.1 B	385.3 AB	1222.3 C	2.8 A	2.3 BC	2.7 B
	YLY900	65.0 A	55.7 E	2340.4 B	792 B	3132.4 BC	90.1 B	76.7 A	925.8 A	373.7 AB	1299.6 A	2.5 C	2.1 C	2.4 C
	XLY900	63.0 C	56.9 C	2072.3 E	833.9 B	2906.2 D	89.2 BC	70.9 ABC	909.5 A	361.5 B	1271.0 AB	2.3 D	2.3 BC	2.3 D
	TYXZ	54.6 E	69.9 A	2251.9 C	1067.5 A	3319.4 A	88.6 BCD	74.8 AB	776.9 C	394.6 A	1171.5 D	2.8 A	2.7 A	2.9 A
	CLYHZ	60.0 D	61.7 B	2281.5 C	769.4 B	3050.9 CD	96.2 A	69.6 BC	919.3 A	321.5 C	1240.4 BC	2.5 C	2.4 B	2.5 C
Treatment	LS	--	59.7 B	--	780.8 B	3039.9 B	--	67.1 B	--	348.8 B	1214.9 B	--	2.3 B	2.5 A
	HS	--	61.1 A	--	1010.9 A	3270.1 A	--	78.6 A	--	397.1 A	1263.1 A	--	2.5 A	2.6 A
Analysis of	V	**	**	**	**	**	**	*	**	*	**	**	**	**
variance	T	--	**	--	**	**	--	*	--	**	*	--	ns	ns
	V*T	--	**	--	**	**	--	ns	--	ns	ns	--	ns	ns

TDW, total dry weight; IP, interception percentage; IPAR, intercepted radiation; RUE, radiation use efficiency; TUE, effective accumulated temperature use efficiency. MS, main season; RS, ratoon season; TS, the total of the two seasons. LS, low stubble; HS, high stubble. Within a column, different lowercase letters indicate statistically significant differences at the *p* ≤ 0.05 level between the two stubbling treatments for each variety. For each stubble height and variety within a column, means followed by different uppercase letters are significantly different according to the LSD test (0.05). Ns, non-significant; ** significant at *p* < 0.01; * significant at *p* < 0.05; --, note.

**Table 3:** Effective accumulated temperature use efficiency, radiation use efficiency, and their related parameters for different ratoon rice varieties in 2023.

Variety	Treatment	TUE (%)		TDW (g m^−2^)			IP (%)		IPAR (MJ m^−2^)			RUE (g MJ^−1^)		
		MS	RS	MS	RS	TS	MS	RS	MS	RS	TS	MS	RS	TS
LY6326	LS	56.3	66.4 b	1503.2	617.6 b	2120.8 b	84.0	48.6 b	681.2	235.9 b	917.1 b	2.4	2.6 b	2.3 b
	HS	56.3	68.5 a	1503.2	904.8 a	2408.0 a	84.0	66.1 a	681.2	301.9 a	983.1 a	2.4	3.0 a	2.4 a
YY4949	LS	60.1	57.1 b	1584.6	476.2 b	2060.7 b	86.0	41.3 b	715.7	196.8 b	912.6 b	2.2	2.4 b	2.2 b
	HS	60.1	58.1 a	1584.6	616.2 a	2200.8 a	86.0	53.3 a	715.7	239.1 a	954.9 a	2.2	2.6 a	2.3 a
YLY900	LS	63.0	51.1 b	1585.9	380.9 b	1966.8 b	86.3	43.2 b	763.3	187.6 b	950.9 b	2.1	2.0 b	2.1 b
	HS	63.0	52.7 a	1585.9	740.7 a	2326.6 a	86.3	67.5 a	763.3	283.6 a	1046.9 a	2.1	2.6 a	2.2 a
XLY900	LS	59.3	56.9 b	1570.7	364.3 b	1935 b	89.9	38.5 b	777.1	173.1 b	950.2 b	2.0	2.1 b	2.0 b
	HS	59.3	58.5 a	1570.7	596.3 a	2167.1 a	89.9	52.8 a	777.1	229.9 a	1007.0 a	2.0	2.6 a	2.2 a
TYXZ	LS	58.3	53.3 b	1688.7	452.0 b	2140.7 b	83.6	46.1 b	712.8	186.8 b	899.6 b	2.4	2.4 b	2.4 b
	HS	58.3	55.9 a	1688.7	626.5 a	2315.2 a	83.6	59.4 a	712.8	233.1 a	945.9 a	2.4	2.7 a	2.4 a
CLYHZ	LS	58.4	53.7 b	1751.0	403.5 b	2154.4 b	85.2	39.4 b	736.7	171.6 b	908.3 b	2.4	2.4 b	2.4 b
	HS	58.4	56.2 a	1751.0	504.8 a	2255.7 a	85.20	46.3 a	736.7	195.3 a	931.9 a	2.4	2.6 a	2.4 a
Variety	LY6326	56.3 D	67.5 A	1503.2 D	761.2 A	2264.4 A	84.5 C	57.3 A	681.2 D	268.9 A	950.1 BC	2.4 A	2.8 A	2.4 A
	YY4949	60.1 B	57.6 B	1584.6 C	546.2 B	2130.8 AB	86.4 B	47.3 B	715.7 C	218.0 BC	933.7 CD	2.3 B	2.5 AB	2.3 B
	YLY900	63.0 A	51.9 D	1585.9 C	560.8 B	2146.7 AB	86.3 B	55.3 A	763.3 A	235.6 B	998.9 A	2.1 C	2.3 B	2.1 C
	XLY900	59.3 B	57.7 B	1570.7 C	480.3 C	2051 B	89.9 A	45.7 B	777.1 A	201.5 CD	978.6 AB	2.0 C	2.4 B	2.1 C
	TYXZ	58.3 C	54.6 C	1688.7 B	539.3 B	2228 A	83.6 C	52.7 A	712.8 C	209.9 C	922.7 CD	2.4 A	2.6 AB	2.4 A
	CLYHZ	58.4 C	55.0 C	1751 A	454.1 C	2205.1 AB	85.2 B	42.8 B	736.7 B	183.4 D	920.1 D	2.4 A	2.5 B	2.4 A
Treatment	LS	--	56.4 B	--	449.1 B	2063.1 B	--	42.8 B	--	192.0 B	923.1 B	--	2.3 B	2.2 B
	HS	--	58.3 A	--	664.9 A	2278.9 A	--	57.6 A	--	247.1 A	978.3 A	--	2.7 A	2.3 A
Analysis of	V	**	**	**	**	**	*	**	**	**	**	**	*	**
variance	T	--	**	--	**	**	--	**	--	**	**	--	**	**
	V*T	--	**	--	**	**	--	*	--	*	*	--	ns	ns

TDW, total dry weight; IP, interception percentage; IPAR, intercepted radiation; RUE, radiation use efficiency; TUE, temperature use efficiency. MS, main season; RS, ratoon season; TS, the total of the two seasons. LS, low stubble; HS, high stubble. Within a column, different lowercase letters indicate statistically significant differences at the *p* ≤ 0.05 level between the two stubbling treatments for each variety. For each stubble height and variety within a column, means followed by different uppercase letters are significantly different according to the LSD test (0.05). Ns, non-significant; ** significant at *p* < 0.01; * significant at *p* < 0.05; --, note.2.4. Correlation analysis between the yield and light and temperature resources.

**Table 4:** Information on varieties for testing.

Variety	Variety Type	Growth Period (Day)	Year of Release	Female Parent	Male Parent
LY6326	Indica	129	2009	xuan69S	WH26
YLY900	Indica	114	2016	Y58S	R900
CLYHZ	Indica	123	2016	C815S	Huazhan
XLY900	Indica	139	2017	Guangxiang24S	R900
TYXZ	Indica	125	2021	Taonong1A	Huanghuazhan
YY4949	indica × japonica hybrid	152	2021	Yongjing49A	F9249

## Data Availability

All data supporting the conclusions of this manuscript are provided within the manuscript.
